# A Peculiar Case of Addison’s Disease

**Published:** 1890-07

**Authors:** A. H. Ohmann-DuMesnil

**Affiliations:** St. Louis, Mo.; Professor of Dermatology in the St. Louis College of Physicians and Surgeons


### A PECULIAR CASE OF ADDISON'S DISEASE.

By A. H. OHMANN-DUMESNIL,

Professor of Dermatology in the St. Louis College of Physicians and Surgeons.

Despite the fact that the literature of Addison's disease is vast and that much has been written upon this peculiar affection, its clinical characteristics and its pathology, each new case which is reported has a certain interest attached to it which is certain to attract more or less attention.

I do not purpose writing an exhaustive paper upon the subject, but merely wish to place upon record a case which proved interesting to me from the fact that a certain symptom presented itself which I have not found recorded in connection with this affection. I wish to state, however, that my search has been by no means exhaustive, and that similar cases might have escaped me in my rather rapid review of a portion of the literature of the subject. This symptom to which I will call attention later on in a more explicit manner is one which I deem of some importance, as it confirms the view that Addison's Disease is primarily and essentially an affection of the sympathetic nervous system. If we can positively determine this to be a fact there may be found some means of arresting the disease, even if the pigmentation cannot be caused to disappear. As the trouble is one which almost invariably terminates fatally, such an advance in the curative management would be a triumph in therapeutics.

That not more is known of Addison's Disease is probably due to the fact that it is an affection not often observed. I have had an opportunity of observing but two cases, in one of which the history was such as we ordinarily find it; whereas, in the other a few points existed which added interest, and which have led me to present a short history and clinical record, which is as follows:

CASE.—On January 6th of this year I was requested by Dr.

W. W. Graves, of this city, to see a case of Addison's Disease. The patient was a man of forty-six who gave the following history: While employed in a grain elevator in Memphis, Tenn., became aware of a general feeling of malaise. This "bad feeling," as he expressed it, became so great that, as soon as he felt able to do so, he left for St. Louis. The beginning of this was about October 15th, 1889. The bronzing of the skin was scarcely noticed at this time, but it progressed steadily. He arrived in St. Louis about December 20th, and a week later it was noticed that he perspired very freely over the whole body, and that the sweat had a most intense and disgusting order. So marked was this that the doors and windows had to be open. The whole house was permeated by this smell which resembled that of carrion. The bromidrosis gradually disappeared and the amount of sweat diminished. The family history was good.

At the time that I examined the man I found him well-developed—perhaps slightly emaciated, but not perceptibly so. Expression of face dull. He was still feeling rather weak although he had improved somewhat of late. He complained of a pain in the pit of the stomach, but pressure exercised in this region did not elicit any pain. He occasionally "bloated," and the tympanites would become so great as to distress him much. He would be unable to put on his clothing, and he stated that it came on suddenly. Pressure on the back over the areas of the kidneys and supra-renal capsules did not elicit any pain. The patient was of a nervous disposition and had always been so.

Inspection showed that the man had a good supply of hair of a dark-brown color—almost black. He stated, however, and was corroborated in this by his sister that, before his present illness, his hair was of a light color—that he was a blonde. The skin of this individual was apparently of normal thickness, no more than the usual moisture being perceptible. In fact, it appeared perfectly normal with the exception of one thing—the color. The whites of the eyes and the mucous membrane of the mouth were devoid of any adventitious pigmentation.

The entire integument, with the exception of that covering the head and the hands, was of a marked brownish bronze tint. The chest seemed to be of a somewhat darker hue than the back. Disseminated throughout the affected area darker macules of various sizes could be observed. The areolæ about the nipples, the axilæ, perineum, and inter-natal fold were darker than the general surface. On the other hand, that portion of the skin lying over the scapulæ was light in color. A few small scars existed upon the chest and these were whitish. On the chest and back their existed numerous white macules of the size and shape of small oats. These were numerous, more particularly upon the chest and upon the forearms. The patient stated that these were small scars from wounds made by the grain which scratched him in the bins of the elevator. While this appeared to be a very plausible explanation to account for the presence of these spots, close inspection failed to furnish any satisfactory evidence of their being scars; and, as excision of a portion of the integument was not permitted, a resort to microscopical examination could not be made in order to obtain confirmatory evidence.

That Addison's Disease is due to some disturbance of the sympathetic nervous system there seems no reason to doubt. The subjective symptoms which are noted are of a nature to point to such a cause and the bronzing of the skin is another sequence dependent upon the same origin. It is pretty well established at the present day that many affections characterized by an increase in the amount of pigment in the skin, are due to disturbed sympathetic innervation. Crocker states that the study of Addison's disease has made it highly probable that, whenever the abdominal sympathetic, especially the solar plexus, is irritated general pigmentation is likely to ensue. Greenhow and McCall Anderson do not look upon the symptoms as dependent upon destruction of the supra-renal capsules but upon the extension of the morbid process to the neighboring parts, especially to the solar plexus and semi-lunar ganglia; the general consensus of authorities of to-day is that there exists involvement of the sympathetic nervous system.

That this process is a severe one, the general prostration and rapidly fatal termination of a certain number of cases show very plainly. But in addition to this, we have anatomical proof furnished in the profound alterations observed in the supra-renal capsules, in the greater number of cases in which necropsy has been performed. Addison was not far from the truth when he stated that the disease was due to these alterations in the capsules; he builded better than he knew. The remarkable investigations and demonstrations of modern investigators have conclusively shown that the supra-renal capsules are ganglia of the sympathetic nervous system directly connected with the solar plexus and semi-lunar ganglia. When we take into consideration the degenerative changes observed in the capsules in some cases, there can be no room left to doubt that the involvement is a very serious one. This discovery also easily accounts for the fact that, in some post mortem examinations, the capsules were found to have suffered no change, a circumstance which would seem to indicate that the process begins most probably in the ganglia situated higher up.

To make a short digression, it is admitted on the part of all authors that functional disturbances of the glands of the skin are also dependent upon disturbed sympathetic innervation. That sympathetic nerves preside over glandular functions is admitted, and the glands of the skin are subject to the general law. This is especially true of those disturbances of function characterized by an increased amount of secretion. Prominent among functional cutaneous diseases is hyperidrosis, of which bromidrosis is merely a variety in the majority of cases. Whether the odor be due to the bacterium fœtidus, to chemical decomposition, or to direct nervous influence as maintained by E. Monin, we need not consider here. The main point to bear in mind is the increased amount of sweat. This, when universal, denotes a more profound implication of the nervous system, than when localized in some particular area.

In the case which I have outlined above, there existed for some little time a general bromidrosis, indicating a rather serious involvement of the sympathetic nervous system. The intensity of the odor also tended to show that the process was a severe one. The sudden onset and as sudden disappearance would further go to support the claim of its nervous origin. The presence of micro-organisms or of chemical decomposition could hardly be claimed, although either one would not militate against the assumption of a neurotic origin for the hyperidrosis.

The fact of the presence of this symptom is interesting on account of its rarity in connection with Addison's Disease, and because, as stated above, it shows the profound involvement of the sympathetic nervous system which must have existed in the case. We find then that the skin is pigmented and that the functions of a set of glands are also changed. In other words, two manifestations of an entirely dissimilar character, and which we are accustomed to study separately, are here found to be coincident, and, beyond any doubt, dependent upon a common origin.

The lack of time and opportunity have prevented my making a study of this question. There is no doubt, whatever, in my mind, that other cases of like nature have occurred, but the importance of the graver malady as well as its comparative rarity completely overshadowed the importance of the symptoms, which is one of frequent occurrence—the bromidrosis.

A feature which was observed, and which might partially account for the fœtid sweat, is the fact noted in the history that there was no pigmentation of the mucous membrane of the mouth. The explanation that this would furnish would be that the energy usually directed to the mucous membrane had been diverted to other channels, or, in other words, the perverted innervation was transferred from the mucous membrane to its external congener, the skin. This process is not an unusual one. We observe it, for instance, in erythema nodosum, in which the eruption rapidly disappears to give way to a bronchitis, and this in turn leaves to be followed by a recurrence of the eruption.

In this short clinical contribution I have merely desired to sketch an interesting condition, and, so far as I know, a peculiar case. While the literature of Addison's Disease is plentiful, there is really little that is tangible. In the light of anatomical investigations, however, we can see the promise of great improvement in the future therapeutical measures to be employed, as well as in the more thorough and intelligent pathological investigations which will be made.

*5 South Broadway.*

